# Design of B‐Cell Multi‐Epitope Subunit Vaccines Against *Glaesserella parasuis* by Reverse Vaccinology: An In Silico and In Vivo Study

**DOI:** 10.1155/tbed/5696948

**Published:** 2026-01-02

**Authors:** Yan Gong, Na Li, Ziheng Li, He Wang, Di Zhang, Hong Chu, Zhichao Lu, Aiqing Jia, Fengyang Li, Liancheng Lei

**Affiliations:** ^1^ State Key Laboratory for Diagnosis and Treatment of Severe Zoonotic Infectious Diseases, Key Laboratory for Zoonosis Research of the Ministry of Education, Institute of Zoonosis, and College of Veterinary Medicine, Jilin University, Changchun, China, jlu.edu.cn; ^2^ Department of Rehabilitation, The Second Hospital of Jilin University, Changchun, China, jlu.edu.cn; ^3^ College of Animal Science and Technology, Yangtze University, Jingzhou, China, yangtzeu.edu.cn; ^4^ Haid Research Institute, Guangdong Haid Group Co., Ltd., Guangzhou, China

**Keywords:** B-cell epitope, Glässer’s disease, *Glaesserella parasuis*, multi-epitope vaccine, reverse vaccinology

## Abstract

Glässer’s disease caused by *Glaesserella parasuis* (GPS) is a severe disease that results in substantial economic losses to the swine industry worldwide. Here we describe a multiepitope vaccine cocktail (MEVC) that was designed using reverse vaccinology and immunoinformatics. The MEVC was comprised of three multiepitope subunits (MESs, designated as TB, 14B, and 24B), which were constructed using 14 B‐cell epitopes predicted from six outer membrane antigens of GPS. The MESs exhibited non‐allergenicity, high antigenicity, solubility, and stability. Predicted secondary and tertiary structures of the MESs were validated and showed strong binding affinity with the swine leukocyte antigen (SLA) by molecular docking. Immune simulation analysis further confirmed robust induction of both cellular and humoral immune responses. Immunization with MESs plus Gel‐01 adjuvant (MEVC) resulted in 80% protection against GPS5 infection in mice, along with significantly increased antigen‐specific IgG levels compared to controls. In conclusion, MEVC is a promising vaccine candidate to protect against porcine Glasser’s disease.

## 1. Introduction

*Glaesserella parasuis* (GPS) is a gram‐negative bacterium that commonly colonizes the upper respiratory tract of pigs [1]. GPS infects weaning piglets and is the etiological agent of Glässer’s disease, a syndrome which is widely spread throughout the world. This disease is characterized by polyarthritis, polyserositis, meningitis, and pneumonia, leading to substantial economic losses to the pig industry worldwide [2]. There are currently 15 serotypes of GPS, among which serotypes 4, 5, and 12 are the most common in sick pigs [3]. Notably, GPS has been isolated frequently and caused coinfections with other important porcine pathogens, including porcine circovirus type 2 (PCV2), porcine reproductive and respiratory syndrome virus (PRRSV), *Streptococcus suis*, and *Actinobacillus pleuropneumoniae* [4–6], resulting in high morbidity and mortality in pigs.

Antibiotics and vaccination are the most widely used strategies for prevention and treatment of Glässer’s disease. However, the extensive use of antibiotics has led to increased antimicrobial and multidrug resistance, which makes many commonly used antibiotics ineffective. Conventional vaccines, including inactivated and subunit vaccines, have been developed to combat Glässer’s disease [7]. Most inactivated vaccines are protective against the same serotype only [8]. On the other hand, subunit vaccines based on effective antigen proteins and more flexible formulas are considered ideal preventive strategies and have the potential to provide protection against different serotypes. Numerous research efforts have focused on the development of subunit vaccines to prevent Glässer’s disease. However, their effectiveness depends entirely on the choice of protein antigen, and novel protective and highly immunogenic antigens need to be identified to improve efficacy.

The use of reverse vaccinology to design multiepitope vaccines (MEVs) is a new strategy to identify efficacious vaccines. Compared with conventional vaccines, MEVs consist of a variety of dominant epitopes derived from multiple target antigens that can simultaneously induce strong cellular and humoral immune responses [9]. Therefore, they overcome the disadvantages of poor immunogenicity, limited immunoprotection, and side effects induced by single antigen‐based vaccines [10]. Thus, MEVs have been extensively studied for the prevention of diverse viral and bacterial infections. However, current research on MEVs against GPS infection is limited, and the protective effects were either not verified in animal experiments or could not provide high protection against GPS infection [11, 12]. Thus, it is crucial to develop MEVs that provide effective protection against GPS infection.

**Figure 1:**
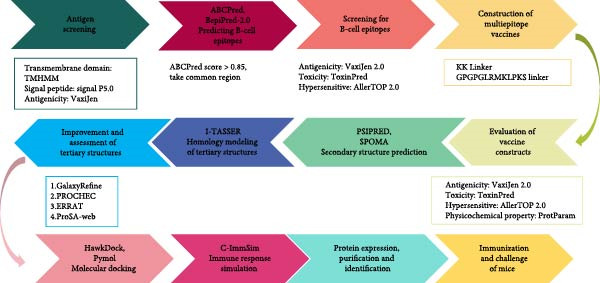
Flowchart for the construction of the MESs in this study.

In this study, an MEV cocktail (MEVC) against GPS infection was developed by an immunoinformatics approach (Figure 1). Dominant B‐cell epitopes from six outer membrane and structural proteins of GPS were screened and tandemly linked using specific linkers, resulting in multiepitope subunits (MESs) TB, 14B, and 24B. The physicochemical properties, secondary and tertiary structures, binding affinities with swine leukocyte antigen (SLA), and immune response were evaluated. Finally, the protective effect of the MEVC was assessed in a mouse infection model. The results suggest that the MEVC is a promising vaccine against porcine Glasser’s disease.

## 2. Materials and Methods

### 2.1. Bacterial Strains and Growth Conditions

All strains used in this study are listed in Table S1. A GPS serotype 5 (GPS5) strain was kindly provided by Prof. Lei Wang (Henan Institute of Science and Technology, Xinxiang, China). GPS strains were cultivated in brain heart infusion (BHI, Hopebio) medium supplemented with 5% horse serum and 20 µg/mL NAD (BHI+) or plated on BHI + agar plates. *E. coli* strains were cultured in Luria‐Bertani (LB; Becton Dickinson) liquid medium or on LB agar plates containing ampicillin (100 µg/mL). Unless specified, cultures were incubated at 37°C with 5% CO_2_.

### 2.2. Antigen Selection for MEV

Six GPS proteins, including four outer membrane proteins, HxuC (GenBank: ACL33614.1), OmpP2 (GenBank: ACS71773.1), TolC (GenBank: WP_207342822.1), and TbpA (GenBank: AAV68905.1), and two uniquely expressed proteins, MreC (GenBank: WP_021111746.1) and PilB (GenBank: WP_015940026.1), during GPS infection were selected to design a B‐cell multi‐epitope vaccine. These proteins play important roles in the colonization and invasion of GPS and have been proposed as potent antigens for the construction of subunit vaccines against GPS infection [13–17]. The protein sequences were obtained from the NCBI database (https://www.ncbi.nlm.nih.gov/genbank/). Moreover, the transmembrane regions, the locations of signal peptides, and the antigenicity of these candidates were predicted using the TMHMM tool (https://www.novopro.cn/tools/tmhmm.html), SignalP5.0 (https://www.novopro.cn/tools/signalp), and VaxiJen V2.0 (https://www.ddg-pharmfac.net/vaxijen/VaxiJen/VaxiJen.html), respectively [18–20]. The signal peptides, transmembrane regions, and intracellular segments were removed, and the proteins with good antigenicity (scoring higher than a default threshold value of 0.4) were investigated further.

### 2.3. B‐Cell Epitope Prediction

The linear B‐cell epitopes of the selected antigens were predicted using the ABCPred online service (https://webs.iiitd.edu.in/raghava/abcpred/index.html) using a threshold of 0.85 and BepiPred‐2.0 using default settings (http://tools.iedb.org/bcell/) [21, 22]. The linear B‐cell epitopes scoring higher than 0.85 (ABCPred) and also predicted by BepiPred‐2.0, were selected for further analysis.

### 2.4. Design of MESs

The antigenicity, allergenicity, and toxicity of all epitopes were evaluated by the VaxiJen v2.0 server at a threshold of ≥0.4, AllerTOP v.21 (https://www.ddg-pharmfac.net/allertop_test/), and ToxinPred (https://webs.iiitd.edu.in/raghava/toxinpred/index.html), respectively [23, 24]. Finally, epitopes with the highest antigenicity, nonhypoallergenic, and nontoxicity were selected to construct the MESs, which were designated as TB, 14B, and 24B. Moreover, KK and GPGPGLRMKLPKS are used as linkers between epitopes [25].

### 2.5. Allergenicity, Toxicity, Antigenicity, and Physicochemical Properties of MESs

The allergenicity, toxicity, and antigenicity of vaccine constructs TB, 14B, and 24B were assessed using VaxiJen, AllerTOP v.21, and ToxinPred, respectively [19, 23, 24]. The physicochemical properties, including molecular weight, theoretical isoelectric point (PI), amino acid composition, estimated half‐life, instability index, aliphatic index, and total hydrophilic mean (GRAVY) of the MESs, were evaluated using the network server ProtParam (https://web.expasy.org/protparam/) [26].

### 2.6. Secondary and Tertiary Structure Prediction and Optimization

The secondary structures of the vaccine constructs TB, 14B, and 24B were analyzed using the PSIPRED (http://bioinf.cs.ucl.ac.uk/psipred/) and SOPMA (https://npsa.lyon.inserm.fr/cgi-bin/npsa_automat.pl?page=/NPSA/npsa_sopma.html) servers, respectively [27, 28]. The tertiary structures of these vaccine constructs were predicted by the I‐TASSER server (https://seq2fun.dcmb.med.umich.edu/I-TASSER/) [29]. The top‐ranked model with the highest *C*‐score was selected as the tertiary structure for the vaccine constructs and was refined by the GalaxyRefine Web server (http://galaxy.seoklab.org/refine) [30]. Global distance test‐high accuracy (GDT‐HA), clash score, Rama favored regions, and root mean square deviation (RMSD) were provided by the GalaxyRefine website, and we detected the chosen structure according to higher percent of Rama favored regions, lower clash score, GDT‐HA closer to 1, and greater RMSD value, respectively. The quality of the refined model was verified by PROCHECK, ERRAT, and ProSA‐web (https://prosa.services.came.sbg.ac.at/prosa.php) [31–33]. The ProSA server evaluated the overall quality of the protein models using the *Z*‐score, an indicator used to check whether the *Z*‐score of the input structure is within the range of scores typically found for native proteins of similar size.

### 2.7. Molecular Docking

The binding affinities between the vaccine constructs and SLA were assessed by molecular docking using the HawkDock server (http://cadd.zju.edu.cn/hawkdock/) [34]. Because of a lack of well‐characterized SLA Class II histocompatibility antigen, the vaccine constructs were docked with the SLA structure predicted by the AlphaFold Database (SLA‐DQ; PDB ID: AF‐P15980‐F1‐v4) [35, 36]. A molecular mechanics/generalized born surface area (MM/GBSA) analysis was used to predict the binding free energy of the vaccine–SLA complexes. Furthermore, the docking results were visualized with Pymol, and the interaction residues between the docked chains were analyzed using PDBsum (https://www.ebi.ac.uk/thornton-srv/databases/pdbsum/) [37].

### 2.8. Immune Response Simulation

The immune response profile of the vaccine constructs was simulated using the C‐ImmSim server (https://kraken.iac.rm.cnr.it/C-IMMSIM/index.php) [38]. The time steps of the three injections in the simulation phase were set to 1, 42, and 84, respectively. The standard time of one injection was 8 h, corresponding to a 14‐day gap between immunizations. The simulation volume is 50, and the simulation step is 1050. All other parameters were set to their default values.

### 2.9. Protein Expression, Purification, and Identification

The recombinant plasmids pET32a::TB, pET32a::14B, and pET32a::24B were constructed after codon optimization and synthesized by Sangon Biotech (Shanghai, China). Plasmids were transformed into *E. coli* BL21 (DE3). Protein expression was induced using 0.1 mM of IPTG for 16 h at 16°C. Bacterial cells were disrupted by sonication and supernatants were collected. Protein purification was performed using a high‐affinity Ni‐NTA resin. In addition, purified proteins were evaluated by SDS‐PAGE and western blotting. After electrophoresis, gels were transferred to a PVDF membrane, followed by blocking with 5% skimmed milk at 37°C for 2 h. A mouse anti‐His (1:3000; Abconal, AE003) and goat anti‐mouse HRP‐IgG (1:2000; Abconal, AS003) were used as primary and secondary antibodies, respectively.

### 2.10. Immunization and Challenge of Mice

All animal experiments were approved by the Institutional Animal Care and Use Committee of Jilin University (Approval Number KT202505005) and were conducted in accordance with the Guidance on the Operation of the Animal (Scientific Procedures) Act 1986 and the Chinese Laboratory Animal Administration Act 1988. All mice were housed in the laboratory animal room and maintained on a 14/10‐h light–dark cycle with food and water ad libitum. For immunization, 100 6‐week‐old healthy female ICR mice (body weight 18 ± 2 g, purchased from Changsheng Biotechnology Co., Ltd., China) were randomly divided into different groups (five mice/group) and immunized with 50 μg recombinant protein mixed with Gel‐01 adjuvant (10%; SEPPIC, France) or inactivated vaccine (Kefuning, China) by multipoint subcutaneous injection on the mice’s backs. Control groups were immunized with PBS (detailed grouping information is listed in Table S2). To enhance the immunogenicity of the vaccine constructs, the outer membrane antigen VacJ of GPS was added [39]. Immunization was every 2 weeks for a total of three doses. Blood was collected from the tail vein of mice at 14 and 28 days after the first dose for detection of antibody levels by ELISA. Alternatively, the mice were challenged with GPS5 (1.5 × 10^9^ CFU, 2 × LD50) by intraperitoneal injection after each dose. Animal death was recorded every 12 h. Mice were euthanized using CO_2_ at the end of the experimental period or if they lost 20% of their maximum body weight for two consecutive days, were immobile, or were found moribund.

### 2.11. ELISA

An indirect ELISA was used to detect the specific antibody levels in mouse sera derived from tail‐vein blood after primary and secondary immunization. In brief, each recombinant protein was diluted separately with coating buffer (0.1 mol/L Na_2_CO_3_–NaHCO_3_, pH 9.6) to 1 μg/mL and coated on an ELISA plate (100 μL/well) at 4°C overnight. After washing, 100 μL of 5% BSA was added to each well and sealed at 37°C for 1 h. Then, the sera in serial dilutions (1:10, 1:10^2^, 1:10^3^, 1:10^4^, 1:10^5^, 1:10^6^) as well as negative control serum were added to the ELISA plate (100 μL/well). After incubation at 37°C for 1 h, goat anti‐mouse IgG HRP (1:5000) was added to each well (100 μL/well) and incubated for another 1 h. The reaction was visualized by the addition of 3,3′, 5,5′‐Tetramethylbenzidine (TMB) reagent (100 μL) and terminated by 2M H_2_SO_4_ (50 μL). Absorbance was detected at OD 450 nm. The cut‐off value was determined using the mean value plus 3SD (standard deviation) of the negative samples.

### 2.12. Statistical Analysis

The experimental data were statistically analyzed using GraphPad Prism (version 9.0, San Diego, CA, USA). Data are presented as mean ± SD from three independent replicates. Differences between the mean values of normally distributed data were assessed using the Kaplan–Meier Log‐Rank test, and one‐way or two‐way ANOVA (Dunnett’s test). A *p*‐value <0.05 was considered to be significant and indicated by “ ^∗^”. A *p*‐value <0.01 was considered to be extremely significant and indicated by “ ^∗∗^.”

## 3. Results

### 3.1. B‐Cell Epitopes Prediction

The protein sequences of MerC, HuxC, OMP2, TolC, TbpA, and PilB were subjected to TMHMM and SignalP5.0 servers to predict transmembrane regions and signal peptides, respectively (Table S3 and Figure S1). Moreover, the amino sequences of the homologous proteins from GPS serotypes 4, 5, and 12 were also compared. All of these proteins are highly conserved among these serotypes (>98% identity). To enhance immunogenicity and protein purification, the signal peptides and transmembrane regions were removed before the B‐cell epitopes prediction using ABCpred and BepiPred‐2.0. A total of 31 B‐cell epitopes were obtained by ABCpred and BepiPred‐2.0, respectively (Table S4). After evaluation of antigenicity, allergenicity, and toxicity using VaxiJen v2.0, AllerTOP v.21, and ToxinPred servers, a total of 13 B‐cell epitopes were selected for construction of the MEVs (Table 1).

**Table 1:** Predicted linear B‐cell epitopes for the construction of the MESs.

Protein	Position	Epitope sequence	ABCpred score	Antigenicity	Allergenicity	Toxicity
Omp2	87–96	RLGSGSKNAA	0.85	1.4547	No	No
	140–155	VGTGGIKYTYEVEESI	0.88	1.1686	No	No
	373–385	IKNKDSDNNKVTD	0.9	1.7714	No	No

TbpA	79–89	SVVEQGRGATT	0.89	1.0347	No	No
	123–134	SGAINEIEYENL	0.86	0.6598	No	No
	282–297	TGEERALPDPVKYKSD	0.93	0.4733	No	No
	320–335	SKQRYDTRDMTYPAYW	0.89	0.5216	No	No
	362–377	DGVAIDYFTEDGVKSS	0.85	0.8616	No	No

HxuC	39–55	AFHWANHTSEKATLNK	0.94	0.6905	No	No

MreC	315–330	DEQKEETVEPEETVNP	0.92	1.1786	No	No
	30–38	GRSSAMIQM	0.9	0.7592	No	No

TolC	104–119	KSSASKGVGSSSNLNS	0.88	1.6	No	No

PilB	295–309	DGLIQTQVNQSINLD	0.87	0.6802	No	No

### 3.2. Construction of the MESs

**Figure 2:**
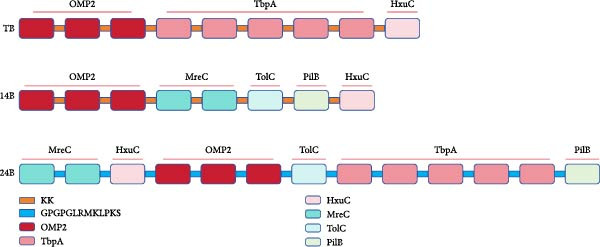
Schematic representation of the vaccine constructs TB, 14B, and 24B. KK and GPGPGLRMKLPKS were used as linkers to connect the B‐cell epitopes of the MESs.

Three vaccine constructs, designated as TB, 14B, and 24B, respectively, were schemed based on the selected B‐cell epitopes, and each contains 8–13 epitopes (Figure 2). To enhance the immunogenicity of the vaccine constructs, a KK or GPGPGLRMKLPKS linker was selected between each epitope. Specifically, TB contained the B‐cell epitopes of OMP2, TbpA, and HxuC, which were connected by KK and consisted of 153 amino acid residues. 14B contained the B‐cell epitopes of OMP2, MreC, TolC, PilB, and HxuC, which were connected by KK, and consisted of 136 amino acid residues. 24B contained the B‐cell epitopes of MreC, HxuC, OMP2, TolC, TbpA, and PilB, which were connected by GPGPGLRMKLPKS and consisted of 332 amino acid residues (Figure 2 and Table S5).

### 3.3. Physicochemical Properties, Antigenicity, Allergenicity, and Toxicity

Subsequently, the physicochemical properties, antigenicity, allergenicity, and toxicity of the three MEVs were evaluated. As shown in Table S6, all three vaccine constructs had higher antigenicity scores than the threshold value, indicating they may trigger a robust immune response. Furthermore, neither of the vaccine constructs was predicted to be potentially allergenic or toxic. The molecular weights of TB, 14B, and 24B were 17.43, 15.04, and 34.02 kDa, respectively. The theoretical PI of the vaccine constructs indicated a slightly basic nature. The instability index for TB and 24B were both below 40, suggesting good protein stability. The instability index for 14B was above 40, suggesting a slightly weak protein stability. The half‐life was 20 h for both TB and 14B in mammals and over 10 h for all the vaccine constructs in *E. coli*. The aliphatic indeces of TB, 14B, and 24B were 51.3, 52.22, and 62.98, respectively, indicating good thermostability of the MEVs. The GRAVY values of TB, 14B, and 24B were below zero, indicating the MEVs are hydrophilic.

### 3.4. Secondary and Tertiary Structure Prediction and Optimization

The PSIPRED and SOPMA servers predicted the secondary structures of the MESs (Figure 3). The vaccine construct TB consists of 41 *α*‐helices (26.62%), nine *β*‐sheets (5.84%), 79 coils (51.3%), and 25 extended strands (16.23%; Figure 3A); 14B consists of 54 *α*‐helices (35.29%), 12 *β*‐sheets (7.84%), 70 coils (45.75%), and 17 extension strands (11.11%; Figure 3B); 24B consists of our *α*‐helices (1.23%), nine *β*‐sheets (2.77%), 230 coils (70.77%), and 82 extended strands (25.23%; Figure 3C). The secondary structures and the topologies of the MEVs were also predicted by PDBsum, and similar results were obtained (Figure S2).

Figure 3Secondary structure of vaccine constructs TB (A), 14B (B), and 24B (C) predicted using the PSIPRED server.

**Figure:**
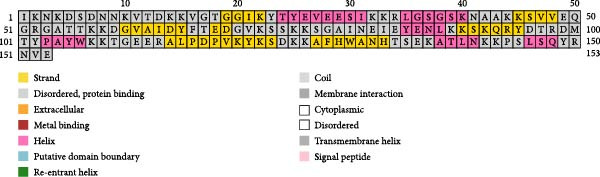
(A)

**Figure:**
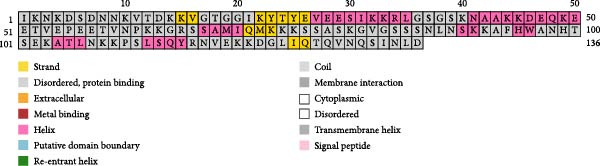
(B)

**Figure:**
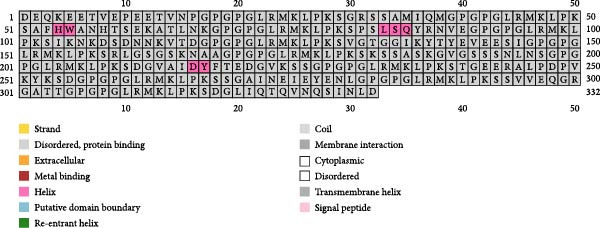
(C)

MESs’ tertiary structures were analyzed through the I‐TASSER server. A total of five models with high *C*‐scores were obtained, and the top‐ranked model with the highest *C*‐score was selected as the tertiary structure for the vaccine constructs that were refined by the GalaxyRefine server. The optimized models are presented in Figure 4A. Subsequently, the models were analyzed by ProSA‐web, ERRAT, and Ramachandran plot. The ProSA‐web showed that the optimized models of TB, 14B, and 24B have a lower *Z*‐score of −2.11, −3.45, and −2.27, respectively (Figure 4B–D). Moreover, the ERRAT showed that the overall quality factors of TB and 14B were both 100 (Figure 4E,F), indicating a good model quality. However, the quality factor of 24B was 86.27 (Figure 4G), indicating an intermediate model quality. The Ramachandran plot analysis indicated that the residues residing in the most favored and allowed regions of the models for TB, 14B, and 24B accounted for 98.5%, 100%, and 96.6%, respectively (Figure 4H–J), all of which were higher than 90% and thus confirmed the overall good quality of the models.

Figure 4Homologous modeling and verification of the three‐dimensional structures of the vaccine constructs. (A) The 3D structures of TB, 14B, and 24B were modeled by the I‐TASSER server and refined by the GalaxyRefine server. The refined models were shown. (B–D) Model quality validation of the models by measuring the *Z*‐scores using the ProSA‐web. The *Z*‐score plot contains *Z*‐scores of all experimental protein chains in PDB determined by NMR spectroscopy (dark blue) and X‐ray crystallography (light blue). (E–G) Overall quality assessment of the models using the ERRAT server. (H–J) Ramachandran plot statistics of the models. Black square, torsion angle of polypeptide; red, secondary structural elements; yellow, favorable area; light yellow, allowable area; white, disallowed area.

**Figure:**
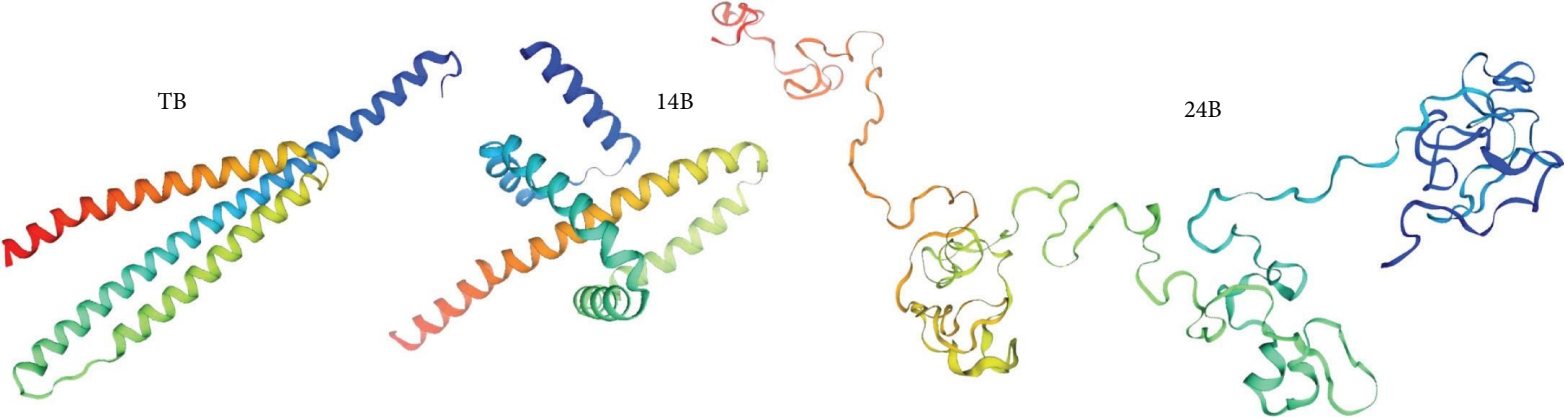
(A)

**Figure:**
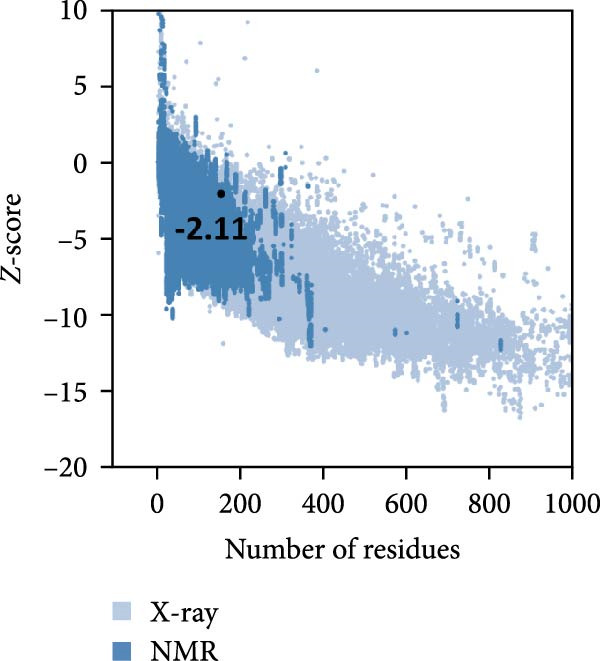
(B)

**Figure:**
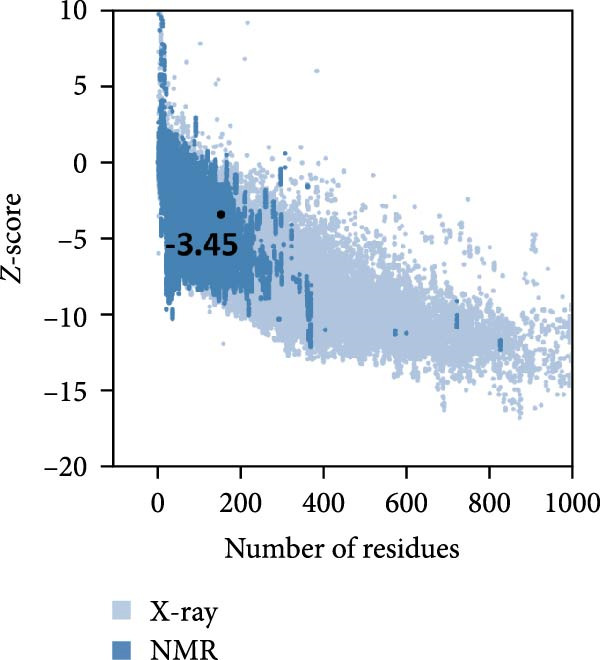
(C)

**Figure:**
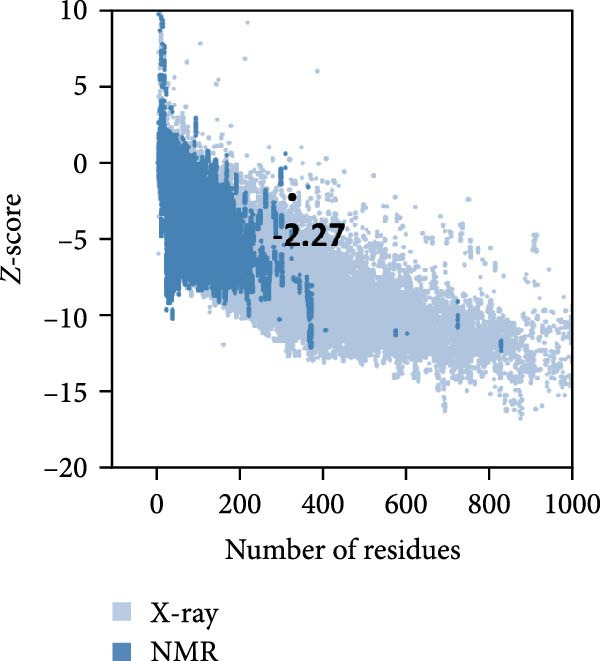
(D)

**Figure:**
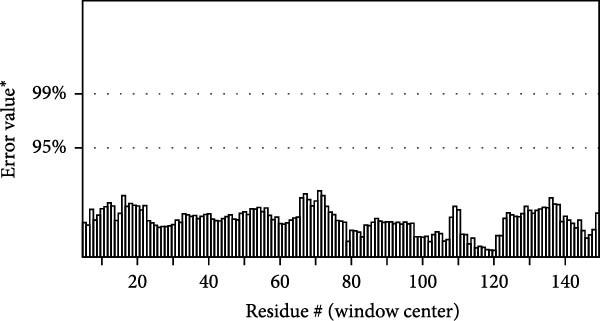
(E)

**Figure:**
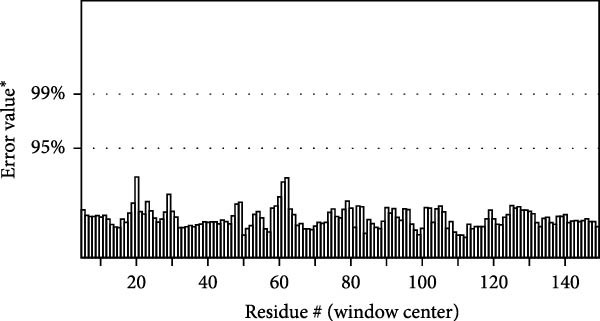
(F)

**Figure:**
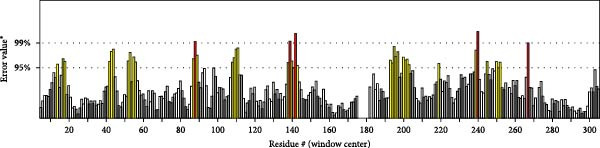
(G)

**Figure:**
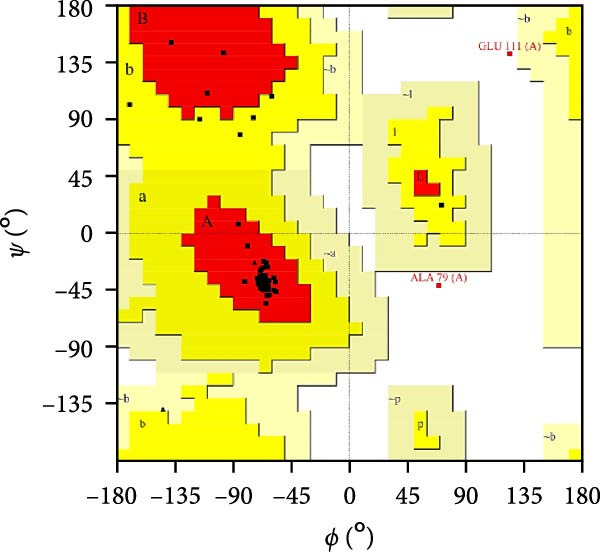
(H)

**Figure:**
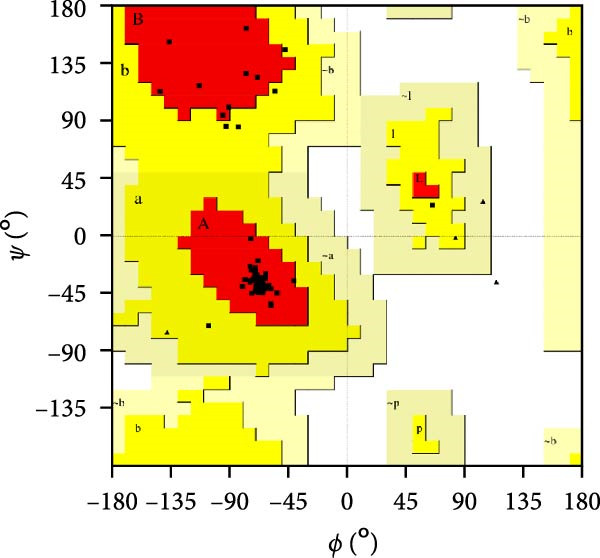
(I)

**Figure:**
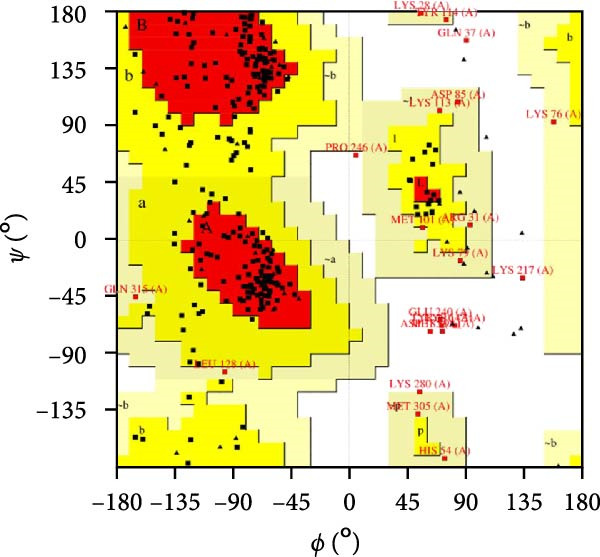
(J)

### 3.5. Molecular Docking of the Vaccine Constructs With SLA‐DQ

To investigate the binding mode between vaccine constructs and SLA‐DQ, molecular docking was performed using the HawkDock server. Ten optimal models were achieved, and the model with the lowest docking score and binding free energy was selected and further analyzed using the PDBsum server. The model displayed good binding affinities for the three vaccine constructs with SLA‐DQ (Figure S3 and Figure 5A–C). A total of 18, 19, and 19 residues from TB, 14B, and 24B, respectively, interacted with 20, 17, and 15 residues from SLA‐DQ (Table S7 and Figure 5A–C). Moreover, there are three salt bridges, three hydrogen bonds, and 194 nonbonded contacts predicted at the binding interface of the TB‐SLA–DQ complex; three salt bridges, six hydrogen bonds, and 151 nonbonded contacts for the 14B‐SLA–DQ complex; three salt bridges, four hydrogen bonds, and 137 nonbonded contacts for the 24B‐SLA–DQ complex (Table S7; Figure 5A–C). Overall, these results indicate the presence of strong interactions between the vaccine constructs and SLA‐DQ.

Figure 5Diagram of the docking model of TB‐SLA‐DQ (A), 14B‐SLA‐DQ (B), and 24B‐SLA‐DQ (C). The dashed box indicates the docking region of each complex, while the right side shows the specific docking amino acid residues and the interactions between each amino acid.

**Figure:**
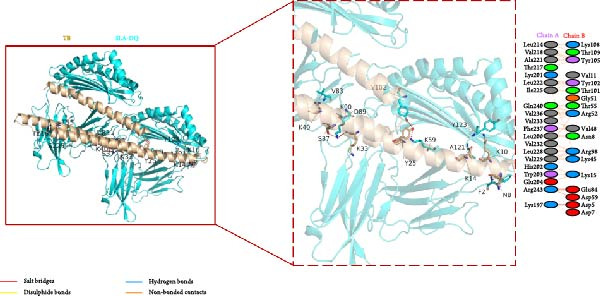
(A)

**Figure:**
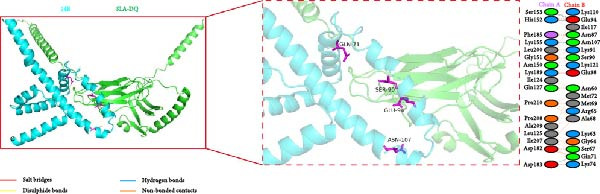
(B)

**Figure:**
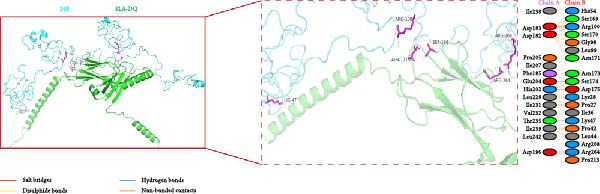
(C)

### 3.6. Immune Simulation

The immune response profiles to the designed vaccine constructs TB, 14B, and 24B were simulated using the C‐ImmSim server, and a broad and robust activation of the immune system postinjection was predicted. Specifically, antibody levels, including IgM, IgG + IgM, and IgG1 + IgG2, were predicted to dramatically elevate after the second and third immunizations of the vaccine constructs, along with a decrease in antigen levels (Figure 6A). Moreover, the total number of B‐cells was predicted to increase remarkably after the second and third immunizations (Figure 6B). Memory B‐cells were predicted to increase after 2 days of the first immunization, accompanied by a decrease in nonmemory B‐cells. The total number of other isotypes, including IgM+ and IgG1 + B‐cells, was also predicted to increase remarkably after the second and third immunizations. The total number of T helper cells (Th) and their isotypes were also predicted to be induced during the whole course of vaccination (Figure 6C). The nonmemory cytotoxic T cells (Tc) were predicted to fluctuate, with an increase, and to reach the highest level at 10–15 days after first immunization (Figure 6D). The total number of NK cells was also predicted to fluctuate during the whole course of vaccination (Figure 6E). Notably, simulation of cytokine response suggested that high levels of IFN‐*γ* and IL‐2 would be induced after immunization of all vaccine constructs, with an increase in TGF‐*β* only after immunization with TB (Figure 6F).

Figure 6In silico immune simulation of the MESs by the C‐ImmSim tool. The graph shows (A) the immunoglobulin levels; (B) B‐cell population; (C) Th cell population; (D) Tc cell population; (E) NK cell population; (F) the expressions of cytokine levels in response to vaccination.

**Figure:**
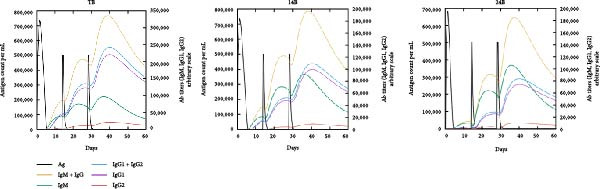
(A)

**Figure:**
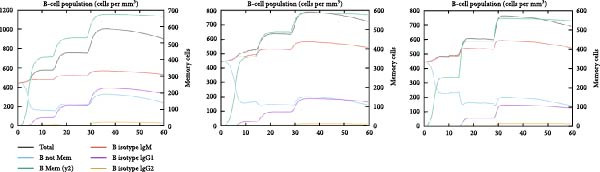
(B)

**Figure:**
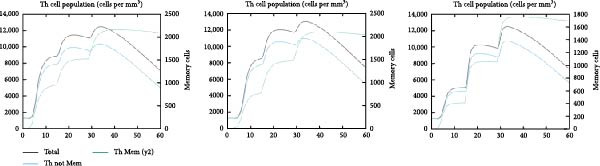
(C)

**Figure:**
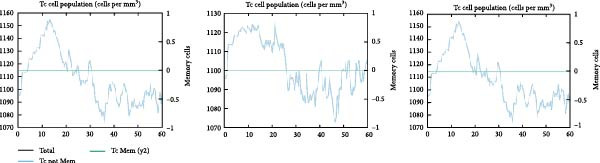
(D)

**Figure:**
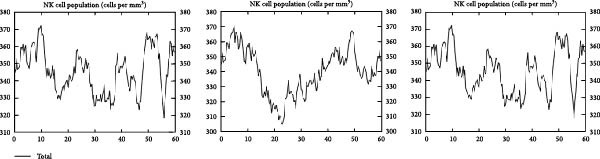
(E)

**Figure:**
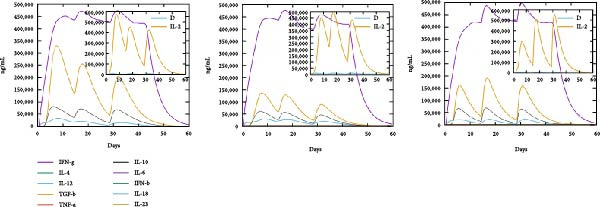
(F)

### 3.7. Purification of Recombinant Proteins

The codon‐optimized sequences of the vaccine constructs TB, 14B, and 24B were successfully cloned into the pET32a vector (Figure S4). Subsequently, the His‐tagged recombinant proteins were expressed, purified, and analyzed by SDS‐PAGE. For all constructs, bands of the expected size were observed (Figure 7A–C). Finally, the recombinant proteins were confirmed by western blotting using an anti‐His monoclonal antibody (Figure 7D).

Figure 7SDS‐PAGE and western blot analysis of purified recombinant TB, 14B, and 24B constructs. Assessment of recombinant TB (A), 14B (B), and 24B (C) expression in *E. coli* BL21 (DE3) by SDS‐PAGE with Coomassie blue staining. M, protein marker; 1, before induction; 2, after induction; 3, supernatant; 4, precipitation; 5, flow‐through; 6, 20 mM imidazole eluent; 7, 50 mM imidazole eluent; 8, 500 mM imidazole eluent. (D) Analysis of antigen reactivity of recombinant TB, 14B, and 24B by western blot. M, protein marker.

**Figure:**
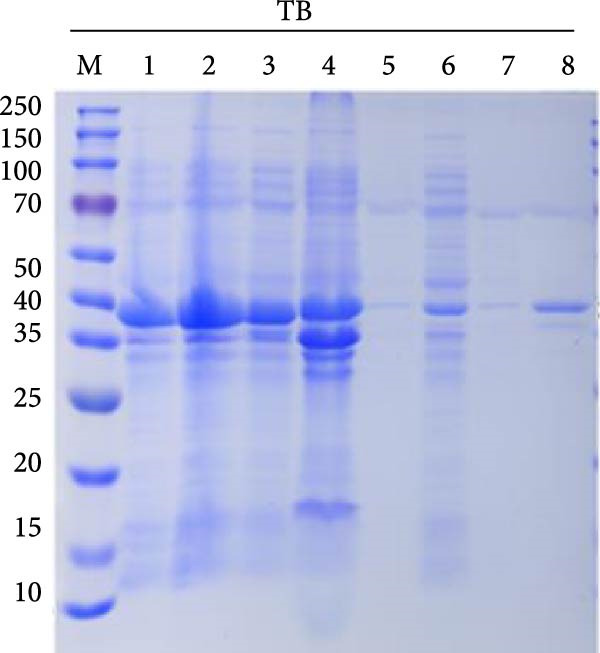
(A)

**Figure:**
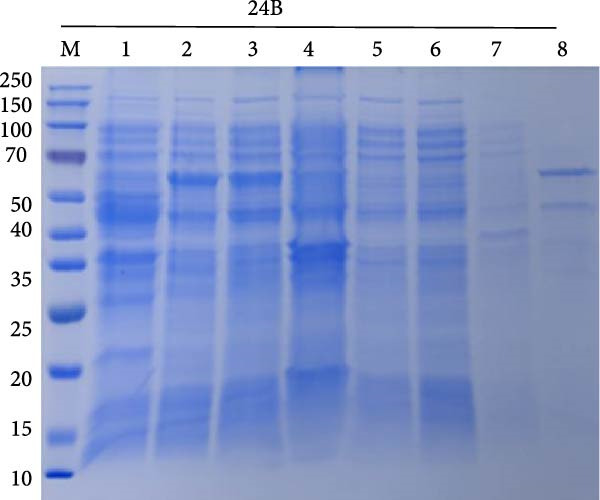
(B)

**Figure:**
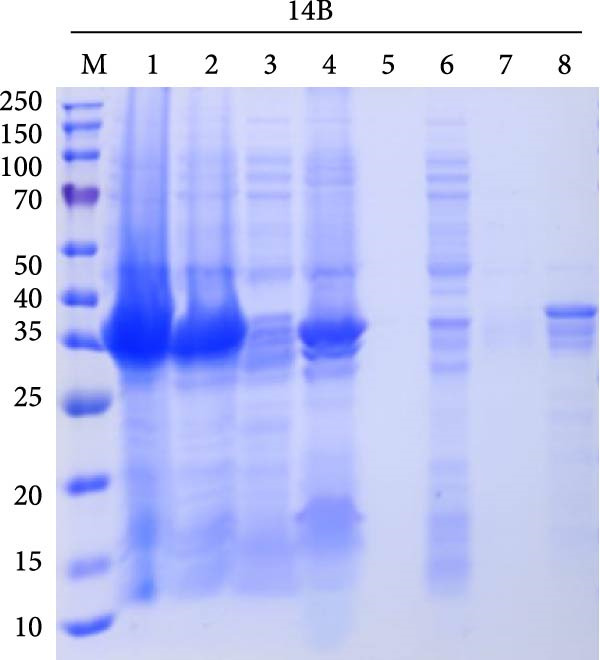
(C)

**Figure:**
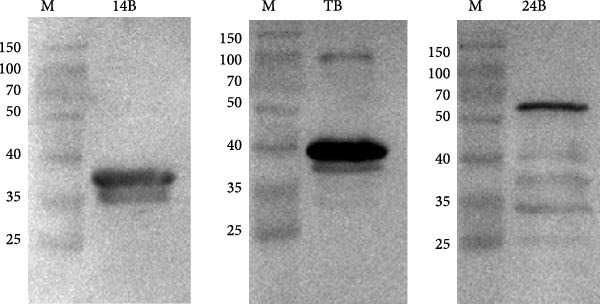
(D)

### 3.8. Evaluation of Immunoprotective Effect in a Mouse Infection Model

The immunoprotective effects of the vaccine constructs TB, 14B, and 24B were evaluated in a mouse infection model. After the GPS5 challenge, the survival of the PBS and adjuvant immunization groups was 40% and 0%, respectively (Figure 8B). All mice administered with inactivated vaccine died after challenge. Mixed immunization with TB, 14B, and 24B, each with VacJ and Gel‐01 adjuvant, increased survival to 60%, 40%, and 40%, respectively (*p* > 0.05). Immunization with VacJ alone resulted in 60% survival. Moreover, immunization with TB combined with VacJ resulted in increased survival from 40% to 60% compared to the control group (*p* > 0.05; Figure 8B). Consistent with these observations, antibody levels in serum increased significantly after the first and second immunizations (*p* < 0.0001; Figure 8C–E).

Figure 8Assessment of the immunoprotective effect of the MEVs in mice. (A) Immunization procedure and blood sampling timeline. (B, F) Survival curves upon challenge with GPS5 in mice immunized with different groups of the MEVs (five mice/group). (C–E, G–I) Antibody levels induced in mice during immunization. Data were expressed as mean ± SD and analyzed using the Kaplan–Meier Log‐Rank test (B, F) and two‐way ANOVA (C–E, G–I). ns, no significance,  ^∗^ *p* < 0.05,  ^∗∗^ *p* < 0.01,  ^∗∗∗^ *p* < 0.001, and  ^∗∗∗∗^ *p* < 0.0001.

**Figure:**
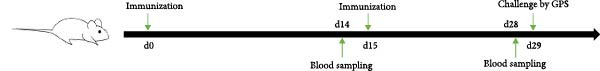
(A)

**Figure:**
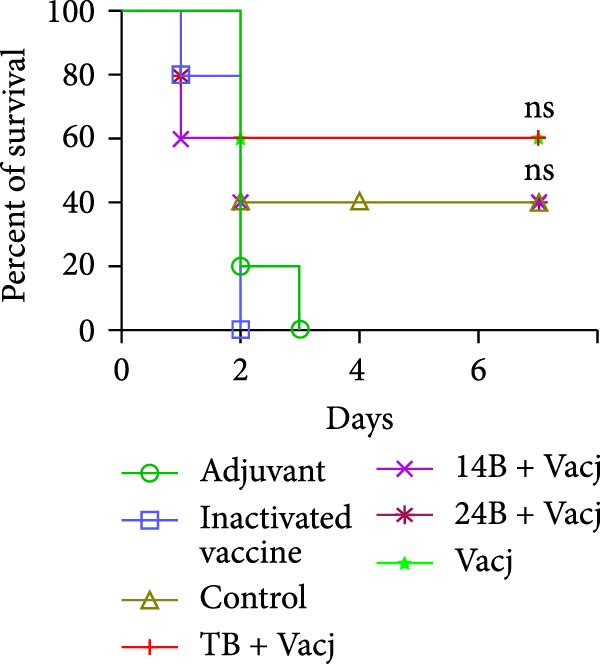
(B)

**Figure:**
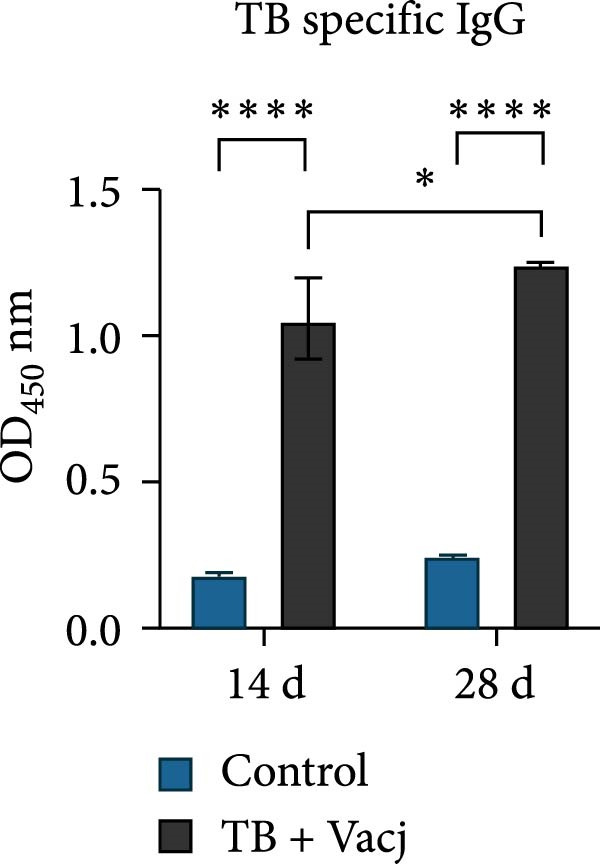
(C)

**Figure:**
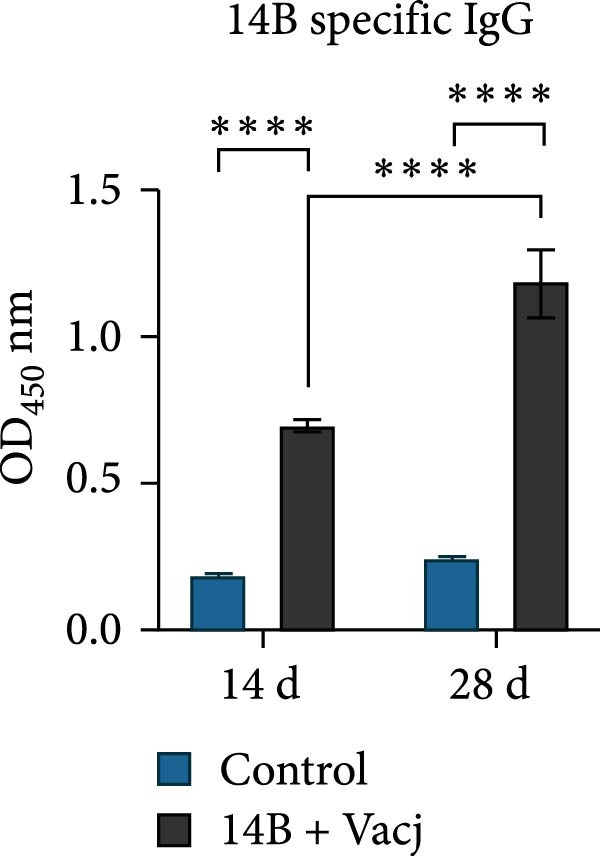
(D)

**Figure:**
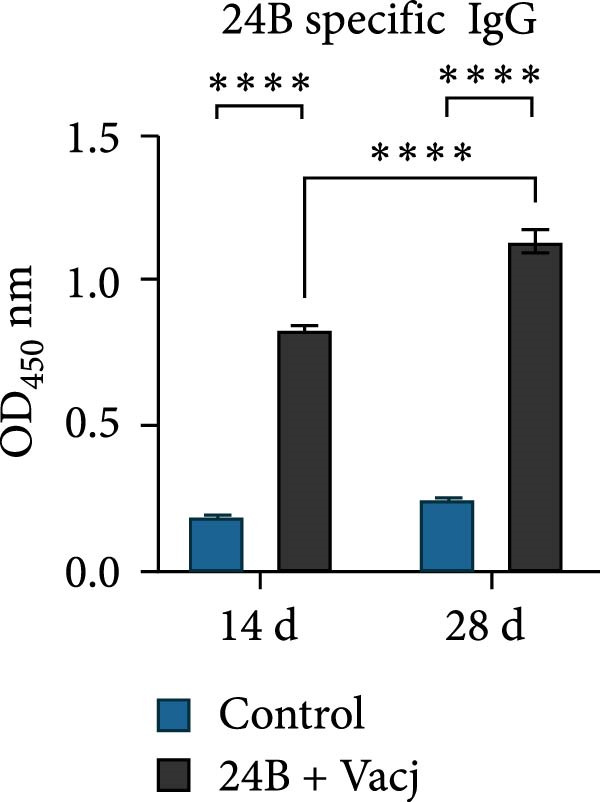
(E)

**Figure:**
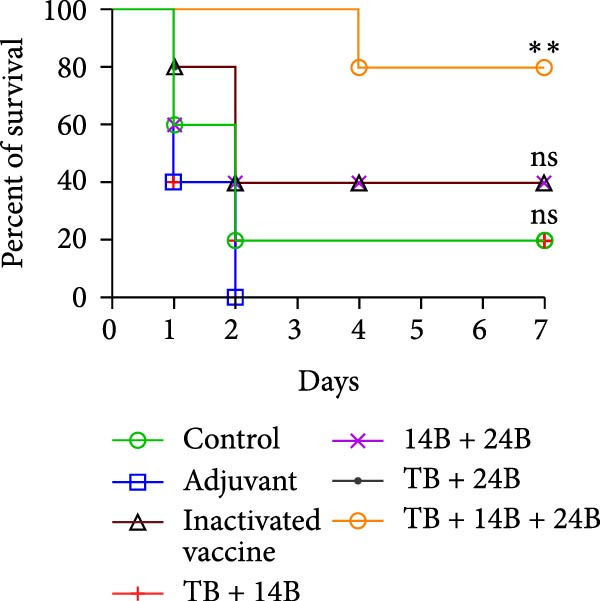
(F)

**Figure:**
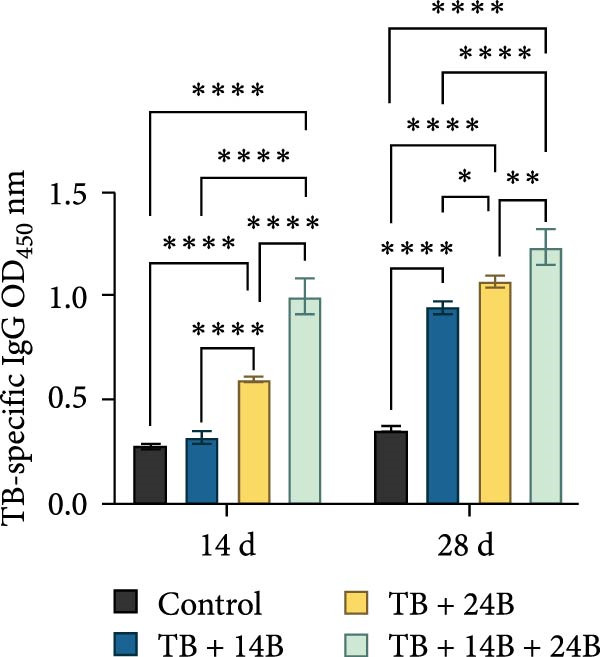
(G)

**Figure:**
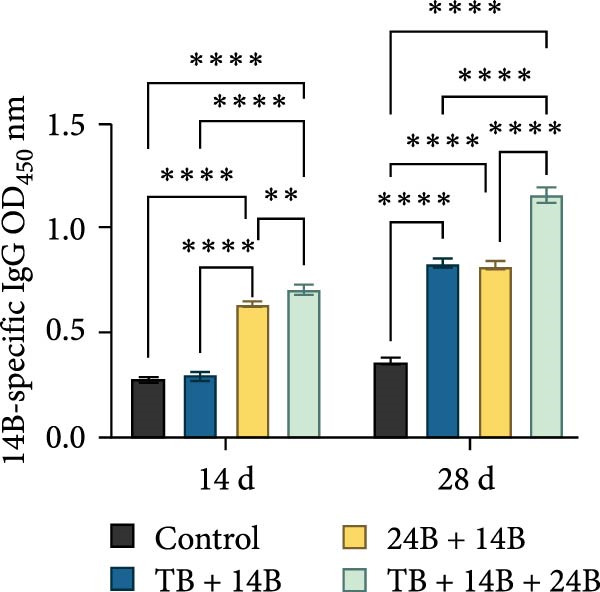
(H)

**Figure:**
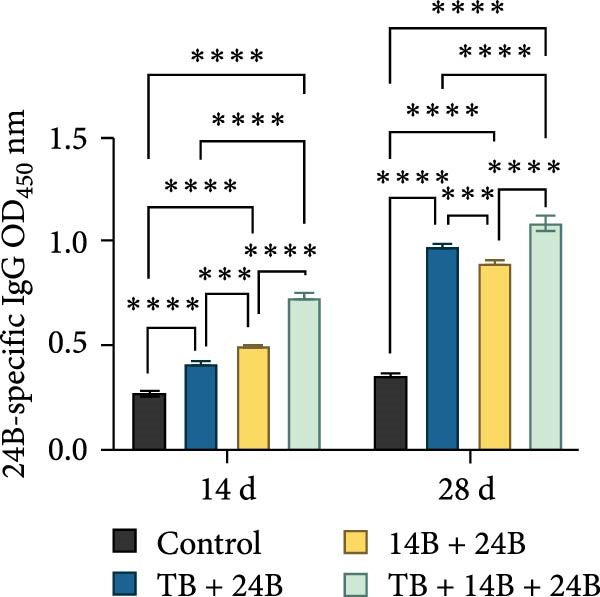
(I)

Next, the vaccine constructs were randomly combined and used to immunize mice. After the GPS5 challenge, survival in the PBS, adjuvant, and inactivated vaccine immunization groups was 20%, 0%, and 40%, respectively (Figure 8F). Combined immunization (TB + 14B + 24B) resulted in increased survival dramatically to 80%, which was significantly higher compared to the adjuvant group (immunization with Gel‐01 and PBS, *p*  < 0.01). Combined immunization with TB + 14B + 24B induced significantly higher antibody levels in sera the first and second immunizations when compared with other groups (*p* < 0.01; Figure 8G–I). Based on these results, the component of the MEVC was determined to be TB + 14B + 24B, with Gel‐01 used as an adjuvant.

## 4. Discussion

Glässer’s disease, caused by GPS infection, is one of the major bacterial diseases in pigs that results in significant economic losses to the porcine industry worldwide. As an emerging pathogen in pig farms, GPS is widely present in healthy pigs, which makes it difficult to control by conventional prevention strategies [2]. Moreover, restriction of antibiotic use and the low efficacy of current commercial inactivated and subunit vaccines have meant that prevention of this disease has been increasingly challenging. In this study, an MEVC *w*as developed by an immunoinformatic approach and its protective effect against GPS infection was determined in a mouse model. Our results demonstrated that MEVC immunization in mice not only induced robust immunogenicity but also conferred significant protection against GPS infection, suggesting it may be used as a promising vaccine against porcine Glasser’s disease.

Antigen selection is a key step in designing MEVs [40]. In this study, B‐cell epitopes were predicted from six antigens, all of which are membrane proteins of GPS. The outer membrane protein P2 (OmpP2) belongs to the porin family and is the most abundant protein in the outer membrane of GPS [41]. It is required for bacterial adhesion and induces inflammatory cytokine expression in porcine alveolar macrophages (PAMs) [42]. TbpA is porcine transferrin binding protein A that is protective against Glässer’s disease [43]. HxuC is the outer membrane receptor for heme acquisition and was identified as a virulence factor of *Haemophilus influenzae* [44]. TolC is an outer membrane lipoprotein that belongs to efflux pumps of the resistance nodulation division (RND) superfamily and participates in the formation of Type I secretion systems of bacterial virulence factors [45]. The immunogenicity and protective efficacy of these proteins have been evaluated in either mice or porcine infection models, and the results suggested they are promising candidate targets for the development of MEVs against Glässer’s disease [14, 16, 43, 46]. MerC is required for maintenance of cell shape in both rod‐ and coccoid‐shaped bacteria [47]. PilB is a motor ATPase that is responsible for the assembly and disassembly, or extension and retraction, of bacterial Type IV pilus, a filamentous cell surface structure that functions in many biological processes, including pathogenesis, biofilm formation, and motility [48]. Interestingly, although no functional studies have been conducted in GPS, the expression of both *merC* and *pilB* was significantly upregulated during GPS infection in mice [15], suggesting a potential role in the regulation of GPS pathogenesis.

To enhance the immunogenicity of the MEVs, mice were immunized with the MEVs in combination with VacJ, a highly conserved and widely distributed outer membrane lipoprotein of *Pasteurella multocida* that is involved in the regulation of cell growth, biofilm formation, and virulence in GPS [49]. Interestingly, mouse survival was not enhanced compared to immunization with VacJ alone (Figure 8B). We reasoned that this may be due to immunodominance, a phenomenon in vaccines containing multiple antigens where the immune system may preferentially mount a strong response to one or several antigenic epitopes while relatively ignoring others [50]. Previous studies have demonstrated that immunization with recombinant VacJ induced insignificant humoral immune responses and provided good immunoprotection against infection by the highly virulent GPS5 strain H46 in guinea pigs [39]. Therefore, the antigenic epitopes of VacJ may possess extremely strong immunogenicity and occupy absolute immunodominance in the combined vaccines. When immunized with TB + VacJ, 14B + VacJ, or 24B + VacJ, the responses of T cells and B‐cells were largely concentrated on VacJ, resulting in the “neglect” or attenuation of responses to other antigens in the MESs. The final protective effect was mainly driven by the immune response against VacJ. Thus, the overall protection rate was similar to that of using VacJ alone, failing to demonstrate the additional effect of the MESs. In addition, it should be noted that survival is only one of the indicators for evaluating vaccine efficacy. Although TB, 14B, and 24B did not seem to make prominent contributions to survival, the impact of MESs may be more significant in other indicators, including bacterial load and pathological lesions of key organs.

Though mouse survival was not altered compared to immunization with VacJ alone, combined immunization of TB, but not 14B and 24B, with VacJ increased survival of mice from 40% to 60%, along with a significant increase in antigen‐specific IgG levels compared to control (Figure 8B,C). This was expected because MEVs were constructed using different antigen epitopes connected with different linkers, and linkers have a critical role in ensuring the functionality of an epitope vaccine [40]. The KK linker is a target for cathepsin B, a key protease in antigen processing [51]. Both the KK and GPGPG linkers prevent the formation of junctional immunogenicity by neoepitopes when individual linear epitopes are connected [52]. The LRMKLPKS linker from the immunoregulatory Ii protein is capable of enhancing the binding of antigenic peptides to MHC class II molecules [53]. Hence, the addition of these linkers contributes to the generation of extended conformations, protein folding, and functional domain segregation to stabilize protein structure and enhance MEV immunogenicity. Conversely, neoepitopes may be formed that have detrimental effects such as induction of unwanted immune responses to irrelevant epitopes resulting in protection failure [54], and may be a reason for the low efficacy of the individual MEVs against GPS infection in the mouse model.

Notably, although the protective efficacy of immunization of the individual MEVs was not substantial, immunization with MEVC (TB + 14B + 24B) conferred a significantly increased survival and robust IgG levels in mice challenged with GPS5 compared to control (Figure 8D–G). This is reasonable because the MEVC contains more epitopes derived from different antigens than a single antigen. Indeed, previous studies have shown that these antigens are all membrane proteins of GPS and are important virulence factors for GPS pathogenesis [13–17]. Therefore, compared with single‐antigen immunization, immunization with the MEVC was more capable of inducing an effective immune response, avoiding the immunodominance interference of a single epitope, and eliciting higher titers of antibodies, thereby better promoting pathogen clearance. Similarly, many studies have shown that combined immunization with different antigens is more effective in preventing disease than a single antigen. For example, Bexsero, a licensed multicomponent vaccine for prevention of meningococcal disease, which comprises conserved, highly immunogenic surface‐expressed protein antigens (fHBP, NadA, and NHBA) with an outer membrane vesicle (OMV), has >90% efficacy against MenB infection in some populations [55].

In this study, the immunogenicity of the MEVs was conducted in a mouse model. It is important to note that mice are not the natural host of GPS, so the pathological processes induced in mice may not fully simulate the complex conditions in the natural host. For example, the typical characteristics of pigs after infection, such as polyserositis, arthritis, meningitis, and pneumonia [2], may not be completely reproduced in the mouse model. However, preliminary experimental results by our group have shown that GPS5 infection in mice caused inflammation in the lungs and liver of mice, along with significantly increased transcription levels of TNF‐*α*, IL‐1*β*, and IL‐6 in these tissues (data not shown), so mice may not be the optimal but can still be used as the experimental model to evaluate the protective efficacy of the vaccine to a certain extent. In addition, the protective efficacy of the vaccine in this study was only tested against GPS5, while its effect on other prevalent serotypes, including GPS4 and GPS12, remains unknown. Finally, the vaccine design in this study was mainly based on B‐cell epitopes to induce humoral immunity. However, effective humoral immunity relies on the activation of CD4+ T helper cells. Although the MESs may contain Th epitopes derived from the same protein, we did not specifically predict or validate them. Nevertheless, our promising results suggest that optimization of epitope sequences, selection of linkers, and B‐cell epitopes will enhance the immunogenicity and efficacy of the MEVs. The ultimate test will be whether optimized MEVs will protect pigs, the natural host of GPS, against Glässer’s disease.

## 5. Conclusions

In this study, three MEVs were constructed by an immunoinformatics approach, and the protective effect of the MEVs was assessed in a mouse GPS5 infection model. Combined immunization with these MEVs dramatically increased the survival of mice after challenge with GPS5. This suggests that these MEVs are promising vaccines against porcine Glasser’s disease.

## Ethics Statement

Animal infection experiments were conducted at the Laboratory Animal Center of College of Veterinary Medicine, Jilin University (Permit Number KT202505005) in accordance with the Guidance on the Operation of the Animals (Scientific Procedures) Act 1986 and the Chinese Laboratory Animal Administration Act 1988.

## Disclosure

All authors have read and approved the final manuscript.

## Conflicts of Interest

The authors declare no conflicts of interest.

## Author Contributions

**Yan Gong:** methodology, data curation, formal analysis, investigation, writing – original draft. **Na Li:** methodology, data curation, formal analysis, investigation. **Ziheng Li and Hong Chu:** methodology, investigation. **He Wang and Zhichao Lu:** validation, software, visualization. **Fengyang Li:** methodology, formal analysis, conceptualization, funding acquisition, supervision, writing – original draft, writing – review and editing. **Liancheng Lei:** funding acquisition, supervision, project administration, resources, writing – review and editing. Yan Gong, Na Li contributed equally to this work.

## Funding

This work was supported by the National Key Research and Development Project Program of China (Grant 2022YFD1800905) and the National Natural Science Foundation of China (Grant 32102670).

## Supporting Information

Additional supporting information can be found online in the Supporting Information section.

## Supporting information


**Supporting Information** Figure S1. Prediction of transmembrane regions and signal peptides of the six proteins MerC, HuxC, OMP2, TolC, TbpA, and PilB using TMHMM (left) and SignalP5.0 (right) servers. Figure S2. Prediction of the secondary structures and the topologies of the MEVs by PDBsum. (A, C, E) The secondary structures of TB, 14B, and 24B; (B, D, F) the topologies of TB, 14B, and 24B. H1, H2, …, helices; *β*, beta turn; *γ*, gamma turn. Figure S3. Molecular docking of the MEVs with swine leukocyte antigen SLA‐DQ. The model with the lowest energy was chosen as the best binding mode. Figure S4. Schematic diagram and construction of plasmid pET32a::TB, pET32a::14B, and pET32a::24B. Table S1. Bacterial strains and plasmids used in this study. Table S2. Experimental groups were used for the evaluation of the immunoprotective effect of the MEVs in mice infection model. Table S3. Prediction of transmembrane regions and signal peptides of the six proteins MerC, HuxC, OMP2, TolC, TbpA, and PilB using TMHMM and SignalP5.0 servers. Table S4. B‐cell epitope prediction using ABCpred and BepiPred‐2.0. Table S5. The final amino acid sequences of TB, 14B, and 24B. The colors of the epitopes and linkers are inconsistent with those in Figure 2. Table S6. Evaluation of the antigenicity, allergenicity, and physical and chemical properties of the MEVs. Table S7. Interface statistics of the docked complexes.

## Data Availability

The data are available from the corresponding author upon reasonable request.
