# Supplementary material for: Design of B‐Cell Multi‐Epitope Subunit Vaccines Against Glaesserella parasuis by Reverse Vaccinology: An In Silico and In Vivo Study
Source: Transbound Emerg Dis. 2026 Jan 2;2026:5696948. doi: 10.1155/tbed/5696948 (PMC12757848; doi:10.1155/tbed/5696948)

**Supplementary tables**

**Table S1.** Bacterial strains and plasmids used in this study

| Bacterial strains | Description^a^ | Source |
| --- | --- | --- |
| *G. parasuis* 5 |  |  |
| *E. coli* DH5α | λφ80d*lacZ* ΔM15 Δ(*lacZYA*-argF)U169  *recA1* *endA1 hsdR17*(rK- mK- ) *supE44*  *thi-1 gyrA relA1* | New England Biolabs |
| BL21(DE3) | F^-^ *ompT hsdSB*(r^-^_B_m^-^_B_) *gal dcm* (DE3) | Novagen |
| Plasmids | |  |
| pET32a | Expression vector with T7 promoter, Amp^r^ | Novagen |
| pET32a::TB | pET32a inserted with synthesized *TB*, Amp^r^ | This study |
| pET32a::14B | pET32a inserted with synthesized *14B*, Amp^r^ | This study |
| pET32a::24B | pET32a inserted with synthesized *24B*, Amp^r^ | This study |
| pET32a::Vacj | pET32a inserted with *Vacj*, Amp^r^ | This study |

^a^Amp^r^, ampicillin resistance

**Table S2**. Experimental groups used for the evaluation of immunoprotective effect of the MEVs in mice infection model.

|  | Experimental groups | Ingredients (200 μL) | Number of mice | Challenging strain |
| --- | --- | --- | --- | --- |
| A | TB + Vacj | 50 μg + 50 μg + 10% Gel-01 | 5 | GPS5 |
|  | 14B + Vacj | 50 μg + 50 μg + 10% Gel-01 | 5 |  |
|  | 24B + Vacj | 50 μg + 50 μg + 10% Gel-01 | 5 |  |
|  | Vacj | 100 μg + 10% Gel-01 | 5 |  |
| B | TB + 14B | 50 μg + 50 μg + 10% Gel-01 | 5 |  |
|  | TB + 24B | 50 μg + 50 μg + 10% Gel-01 | 5 |  |
|  | 14B + 24B | 50 μg + 50 μg + 10% Gel-01 | 5 |  |
|  | TB + 14B + 24B | 33 μg + 33 μg + 33 μg + 10% Gel-01 | 5 |  |
| C | TB + 14B + 24B (1:1:1) | 33 μg + 33 μg + 33 μg + 10% Gel-01 | 5 |  |
|  | TB + 14B + 24B (1:3:1) | 20 μg + 60 μg + 20 μg + 10% Gel-01 | 5 |  |
|  | TB + 14B + 24B (1:1:3) | 20 μg + 20 μg + 60 μg + 10% Gel-01 | 5 |  |
|  | TB + 14B + 24B (3:1:1) | 60 μg + 20 μg + 20 μg + 10% Gel-01 | 5 |  |
|  | Adjuvant group | PBS with 10% adjuvant | 5 |  |
|  | Inactivated vaccine group | 200 µL inactivated vaccine | 5 |  |
|  | Control group | 200 µL PBS | 5 |  |

**Table S3.** Prediction of transmembrane regions and signal peptides of the six proteins MreC, HxuC, OMP2, TolC, TbpA and PilB using TMHMM and SignalP5.0 servers.

| Protein | Transmembrane | Signal Peptide | The aa sequences after removing the transmembrane regions and signal peptides |
| --- | --- | --- | --- |
| TbpA | NO | 1-23  MKNKLNLISLALLSLFAVQSYA | AEQAVQLNDVYVTGTKKKAHKKENEVTGLGKVVKTSDSLSKEQVLGMRDLTRYDPGISVVEQGRGATTGYSIRGVDRNRVGLALDGLPQIQSYVSQYSRSSSGAINEIEYENLRSIQISKGASSSEFGSGSLGGSVQFRTKEVSDIIKPGQSWGLDTKSAYSSKNQQWLNSLAFAGSHNGFDALVIYTHRDGKETKAHKDAESRSKSIQRVALADNNPQGSNWFKVKNDCPSLNCEPRQQVGVSYDGQKYITEQLSGKEYTGEERALPDPVKYKSDSWLVKLGYSLSPKHYVAGVYEHSKQRYDTRDMTYPAYWQLSDLKNSDTWYSMDNAKGLYRDNALDGVAINYFTDGVVKSSKGLRWAKARFIDEWHTRDRLGALYRYTNQDENRLIDSLSLSFDQQKIDLSTRLRENNCSEYPTVDKNCRATLDKLWSSTKNEQSSYEEKHDTIQLSLDKTIQTGLGKHQLNMLLGSDRFNSTLKRHEILSEFSVGTWHRISGNGYQNNPYIYELKDQKIYAQNVCDYTGTIAGKADCATSKIKGHNHYIALRDNFAITKYLDIGLGYRFDKHKFRSTHRWANQGDYKNSAWNIGIVAKPTSFLSLSYRASSGFRVPSFQELFGLRYDGARAGSSDAYQKTEKLSPEKSLNQEVAATFKGDFGIVEVSYFKNDYKQLIAPAERMHQTQSMINYFNVQDIKLDGINLIGKLDWNGVFDKILEGIYTTLAYSKMRVKEVKNYPGYMNIRTPLLDTIQPARYVVGVGYDQPDEKWGVNLTMTHSSGKNPDELRGNEQVGFANYERTATKKRTRSWHTFDLTGYITPWKHITVRAGVYNLMNYRYTTWESVRQSSLNAIHQHTNVKDYARYAAPGRNYVLSVEMKF |
| MreC | 13-30 | NO | MKPIFAKAPPIGVRLFFAIAISISLILLDGRSSAMIQMRNVLETAVSGLYYFANTPRTVLDGVSNNFIDSQKLQVENRVLKEQLREKNADLLLLDQLKVENQRLRLLLSSPLRQDEYKKVAEVLTAEMDAYRQQVVINQGQSDGAFVGQPVIDERGVVGQVISVGKNSSRVLLLTDVTHAIPVQILRNDVRAIANGNGHSNELILDNLPRTVDIGKGDVLVTSGLGGRFPEGYPVAVIETVQNDGSNHFARATARPLASLDRLRYLLLLWPTSEEIRNASSLSPQDVRDAVEERRKSLNPLDRLKNSKKEQVSSDEQKEETVEPEETVNPATQPEVDNGINHSTEQGAE |
| pilB | NO | NO | MSQYTVCEQQTERIFDISPSRWQQNCEEKELLLRYLAVPVQETEHKLWLAVDDENNLTACEIFAFMTHKQIEPVVIASDELKYLLNALSPEQQPFYEESELAFAEQEQEQLNLSDPIIQLLDNLFKFCLAQNASDIHIEPRKQKLIIRLRIDGVLHLYKSLSIQLASRLISRIKLLAKLDISELRQPQDGQFSFTTALADTLDFRVSSLPTIYGEKLVLRLQKNKPTSFDFLQLGFNPQQQTTLLNALKQPQGLILVTGPTGSGKSITLYSALNYLNQMDKHILTAEDPVEIEIDGLIQTQVNQSINLDFSQLLRTFLRQDPDIIMLGEIRDEESAKMALRASQTGHLVLSTLHTNDALLAVERLLQLGIQEYEIQNSLLLVIAQRLVRKLCKKCCGTGCDECYQGYKGRIGIYQCLSRTAKKFDKQTAYLDFSSLRESAKQKIEQQLTNEIEVDRVLGDE |
| HxuC | NO | 1-21  MRLSKIYTALFLSLPTVSLA | DSNVSAELAEISVIASRDAFHWANHTSEKATLNKNQLLTQQPISTADAIKRITNVDIAGGSRGIAQKPIIRGLSGNRVVQVIDGVRQNFDLSHRGSYFLPMALTQEIEVIKGPTSTLWGSGALGGIVAIRTPNALDLLKDNQQIGAKIHQGYQSANSLSETDVSVFAANDKFDGLVSGFYNHADDLRLADSKKLVDSRYTQKGGLFKFGWQINDENRLELSHRLSHFEQIAPTNNEVAEEFTNQDVRNLIRNWHRNNPTASFAEMTRFYKGLTSVLGSVSYRAKQKITDQSSALHYYFNPKDNDYINSQLTLYRNSTKEKEQRIASGLQDQTKLSTMGLNIRNSTDLNWLALNYGVDFISDRVVTQREKNATTPFRPNNYDAKSKNSAFYLLTHIPLWNERIIVSPSVRYDHFKTQSDEAKYSASRWSPSIAATWKATNWLDLSTRYNKAFRTPSMQERFVSGSHFGTNLMGQSFNNTFVSNPNLRPETAKNKEIAAKVHFDNLFVHQDKFIFNAAYFQNDVKDFIHLDIFRASQRERIPSLSQYRNVENARLTGYELEFAYQQERLALGLNYAQTKGKNRQTQEALSNIPANKLGFTIDYALVPQTFMIGANITHYASQKRVPKTHAMTYPSYTLTDLKASYTPSGEWRNLRLDMMIENVFDKKFQPAFSLMEGAGRNIKLNASYQF |
| TolC | NO | 1-19  MKVSKLTFTLLSALVLSA | CTNMSNDGSLQKAQQDYQQYQDITKQYQINEQWWLGYNDTQLNRLVETALANNINLAKTAISVNKALYNANLVGANLVPTFTASEKSSASKGVGSSSNLNSTGTSSIGHQISLNLSYTLDLWGRLRDTASAAEWEHKATQKDLQAARLSLINAVVSSYYNLAYYQDAIRITQQSIKSYEQINRILSNKFKQGLIDQLSVDQSMQAVLSAQNTLINLQSAQKSSEQVLRNLLNLKPNEPLVVNYPSILKVKLQDVDVNVPVSTIANRPDVLASLQRLQGAFKNLTATENSWFPTVTLGGSLTGSSTKFNNTSDNPVAGGVVSFDLPFLDWNRVQNNIKISEESYKLAKLNYEQTVTTALNEIDTYYSTYQLSKSGYANLQKKYEYDRKISGYYKNRYDQGISELREWLSAMNTERSSELSLLENKYTLLKNENAIYQAMAGKYRK |
| Omp2 | NO | 1-20  MKKTLVALAVATFAASASA | VTIYENEGTKVDFDGQLRLLLEKQASKEKAKSSTDGHTNLKNNSSRFGISIKHNINENLYGFGRYETRLGSGSKNAAKWGDVTTDEAYVGLGGYGHEISFGKQAVIGDSIGQAGFDKVYGVGTGGIKYTYEVEESITVDNKQGTFKYSAAQEGFDILTQSSDSAINYTYTGIEGLTLGANYNVANEREKADVKVDSIKSGFGLGAKYTAKIAESQSVTVAAGYTHDDYKSGSVKLKGKFVEAGGKSTDHTYTEKPFNKKDKDGVYFGLKYVNAPFTVAVDGGHGVVKTDDVKEKINFVRTGARFDVTPKSGVYGNYSYGTYKVEDFKVTAHQFMLGADYKLHKQVVTFVEGRLIKNKDSDNNKVTDKALGVGLRVLW |

**Table S4**. B cell epitopes prediction using ABCpred and BepiPred-2.0.

| Protein | Location | Epitope sequence | ABCpred score | Antigenicity | Allergenicity | Toxicity |
| --- | --- | --- | --- | --- | --- | --- |
| TbpA | 79-89 | SVVEQGRGATT | 0.89 | 1.0347 | NO | NO |
|  | 123-134 | SGAINEIEYENL | 0.86 | 0.6598 | NO | NO |
|  | 282-297 | TGEERALPDPVKYKSD | 0.93 | 0.4733 | NO | NO |
|  | 320-335 | SKQRYDTRDMTYPAYW | 0.89 | 0.5216 | NO | NO |
|  | 362-377 | DGVAIDYFTEDGVKSS | 0.85 | 0.8616 | NO | NO |
|  | 398-413 | GALYRYTDQDGNRLID | 0.86 | 0.3784 | Yes | NO |
|  | 511-526 | VGTWHRIRGNGYKDTP | 0.93 | 0.2829 | NO | NO |
|  | 533-548 | DQAIYSKNECDYSGTI | 0.88 | 0.5666 | Yes | NO |
|  | 750-765 | VKEVKNYQGYMNIRSP | 0.85 | 0.4309 | Yes | NO |
| HxuC | 39-55 | AFHWANHTSEKATLNK | 0.94 | 0.6905 | NO | NO |
|  | 309-324 | DQSSALHYYFNPKDND | 0.92 | 0.8513 | Yes | NO |
|  | 391-403 | ATTPFRPNNYDAK | 0.9 | 0.8173 | Yes | NO |
|  | 560-569 | PSLSQYRNVE | 0.92 | 0.5076 | NO | NO |
|  | 689-704 | AFSLMEGAGRNIKLNA | 0.89 | 1.2956 | Yes | NO |
| Omp2 | 49-64 | AKSSTDGHTNLKNNSS | 0.85 | 1.5768 | Yes | NO |
|  | 87-96 | RLGSGSKNAA | 0.85 | 1.4547 | NO | NO |
|  | 140-155 | VGTGGIKYTYEVEESI | 0.88 | 1.1686 | NO | NO |
|  | 172-183 | GFDILTQSSDSA | 0.86 | 0.8969 | Yes | NO |
|  | 243-253 | THDDYKSGSVK | 0.89 | 1.7989 | Yes | NO |
|  | 263-278 | GKSTDHTYTEKPFNKK | 0.88 | 1.0129 | Yes | NO |
|  | 373-385 | IKNKDSDNNKVTD | 0.9 | 1.7714 | NO | NO |
| TolC | 26-41 | GSLQKAQQDYQQYQDI | 0.85 | 0.3864 | NO | NO |
|  | 104-119 | KSSASKGVGSSSNLNS | 0.88 | 1.6719 | NO | NO |
|  | 317-331 | SLTGSSTKFNNTSDN | 0.93 | 1.3898 | Yes | NO |
|  | 405-420 | KISGYYKNRYDQGISE | 0.9 | 0.6902 | Yes | NO |
| PilB | 295-309 | DGLIQTQVNQSINLD | 0.86 | 0.6802 | NO | NO |
|  | 89-104 | SPEQQPFYEESELAFA | 0.85 | -0.097 | NO | NO |
| MreC | 285-300 | QDVRDAVEERRKSLNP | 0.93 | 0.6482 | Yes | NO |
|  | 315-330 | DEQKEETVEPEETVNP | 0.92 | 1.1786 | NO | NO |
|  | 30-38 | GRSSAMIQM | 0.9 | 0.7592 | NO | NO |
|  | 58-71 | TVLDGVSNNFIDSQ | 0.85 | 0.4050 | Yes | NO |

**Table S5**. The final amino acid sequences of TB, 14B, and 24B. The colors of the epitopes and linkers are in consistent with those depicted in Fig. 2.

| Epitope protein | Sequence |
| --- | --- |
| TB | IKNKDSDNNKVTDKKVGTGGIKYTYEVEESIKKRLGSGSKNAAKKSVVEQGRGATTKKDGVAIDYFTEDGVKSSKKSGAINEIEYENLKKSKQRYDTRDMTYPAYWKKTGEERALPDPVKYKSDKKAFHWANHTSEKATLNKKPSLSQYRNVE |
| 14B | IKNKDSDNNKVTDKKVGTGGIKYTYEVEESIKKRLGSGSKNAAKKDEQKEETVEPEETVNPKKGRSSAMIQMKKKSSASKGVGSSSNLNSKKAFHWANHTSEKATLNKKPSLSQYRNVEKKDGLIQTQVNQSINLD |
| 24B | DEQKEETVEPEETVNPGPGPGLRMKLPKSGRSSAMIQMGPGPGLRMKLPKSAFHWANHTSEKATLNKGPGPGLRMKLPKSPSLSQYRNVE GPGPGLRMKLPKSIKNKDSDNNKVTDGPGPGLRMKLPKSVGTGGIKYTYEVEESIGPGPGLRMKLPKSRLGSGSKNAAGPGPGLRMKLPKSKSSASKGVGSSSNLNSGPGPGLRMKLPKSDGVAIDYFTEDGVKSSGPGPGLRMKLPKSTGEERALPDPVKYKSDGPGPGLRMKLPKSSGAINEIEYENLGPGPGLRMKLPKSSVVEQGRGATTGPGPGLRMKLPKSDGLIQTQVNQSINLD |

**Table S6.** Evaluation of the antigenicity, allergenicity, physical and chemical properties of the MEVs.

| Epitope protein | TB | 14B | 24B |
| --- | --- | --- | --- |
| Antigenicity | 0.9446 | 1.2854 | 0.9051 |
| Allergenicity | NO | NO | NO |
| Relative molecular weight (kDa) | 17.43 | 16.99 | 34.02 |
| Theoretical PI | 9.66 | 9.54 | 9.87 |
| Half-life | 20 h (mammalian reticular cells, *in vitro*); > 30 min (yeast, *in vivo*); > 10 h (*E. coli*, *in vivo*) | 20 h (mammalian reticular cells, *in vitro*); >30 min (yeast, *in vivo*); >10 h (*E. coli*, *in vivo*) | 1.1 h (mammalian reticular cells, *in vitro*); 3 min (yeast, *in vivo*); >10 h (*E. coli*, *in vivo*) |
| Instability index | 27.18 | 41.27 | 37.99 |
| Aliphatic index | 51.3 | 52.22 | 62.98 |
| GRAVY | -1.290 | -1.328 | -0.762 |

**Table S7**. Interface statistics of the docked complexes.

| Models | TB-SLA-DQ | | 14B-SLA-DQ | | | 24B-SLA-DQ | |
| --- | --- | --- | --- | --- | --- | --- | --- |
| Chains | SLA-DQ | TB | SLA-DQ | 14B | SLA-DQ | | 24B |
| No. of interface residues | 20 | 18 | 17 | 19 | 15 | | 19 |
| Interface area (A^2^) | 1360 | 1322 | 972 | 955 | 984 | | 869 |
| No. of salt bridges | 3 | | 3 | | 3 | | |
| No. of disulphide bonds | 0 | | 0 | | 0 | | |
| No. of hydrogen bonds | 3 | | 6 | | 4 | | |
| No. of non-bonded contacts | 194 | | 151 | | 137 | | |

**Supplementary figures**

**Figure S1.** Prediction of transmembrane regions and signal peptides of the six proteins MreC, HxuC, OMP2, TolC, TbpA and PilB using TMHMM (left) and SignalP5.0 (right) servers.


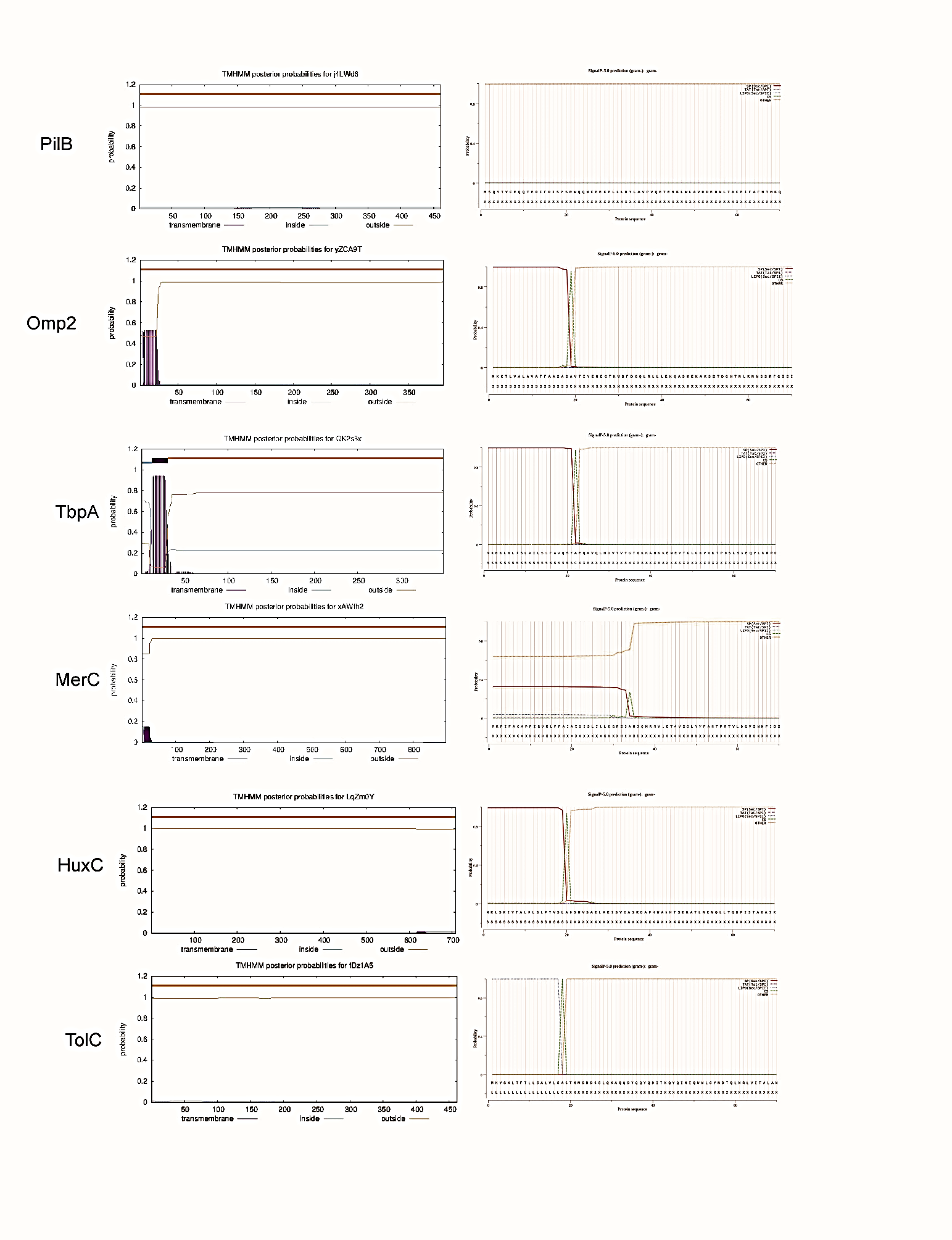


PilB

Omp2

TbpA

MreC

HxuC

TolC

**Figure S2**. Prediction of the secondary structures and the topologies of the MEVs by PDBsum. (A-C) the secondary structures of TB, 14B and 24B; (D-F) the topologies of TB, 14B and 24B. H1, H2, …, helices; β, beta turn; γ, gamma turn.


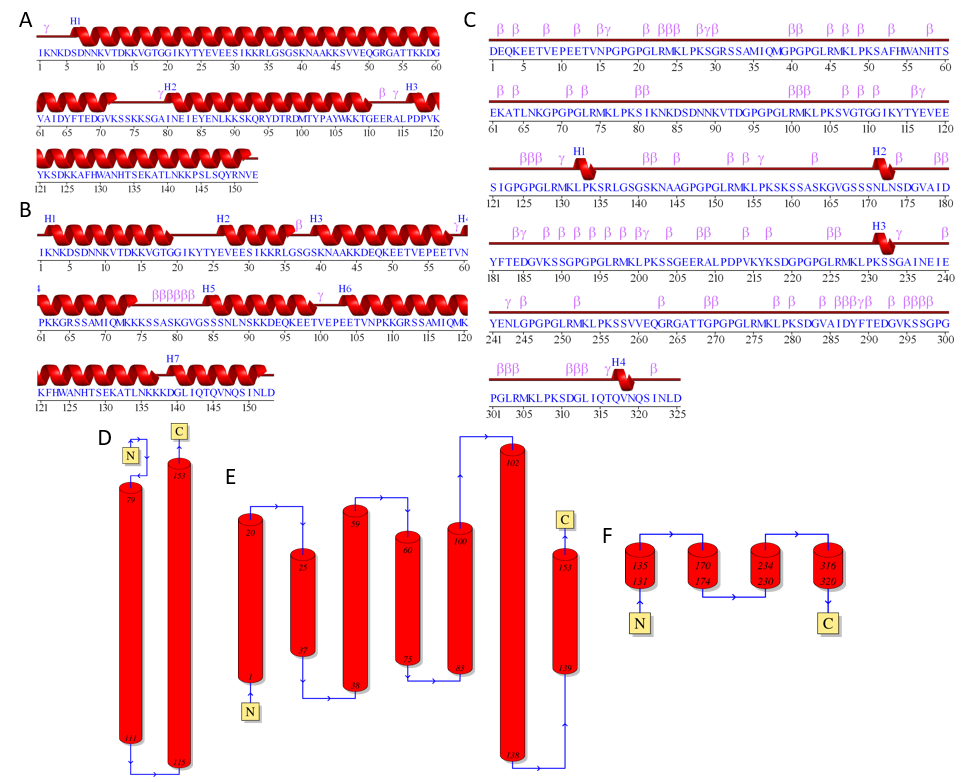


**Figure S3**. Molecular docking of the MEVs with swine leukocyte antigen SLA-DQ. The model with the lowest energy was chosen as the best binding mode.


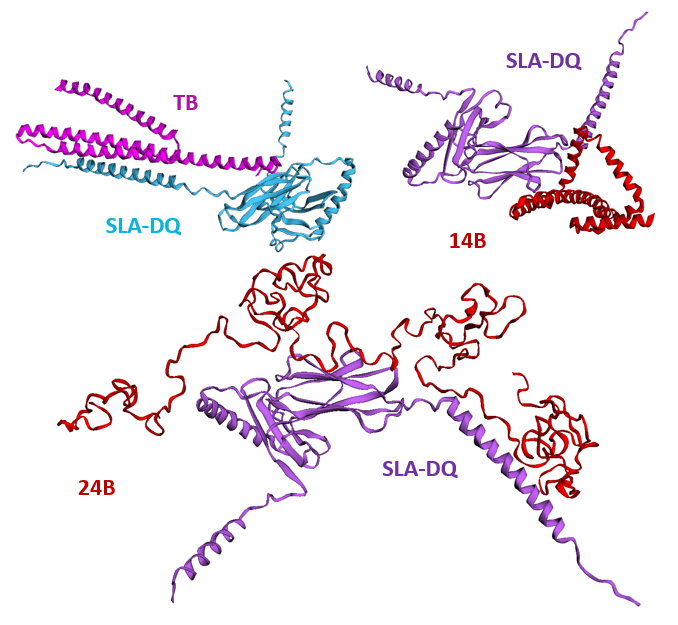


**Figure S4**. Schematic diagram and construction of plasmid pET32a::TB, pET32a::14B and pET32a::24B.


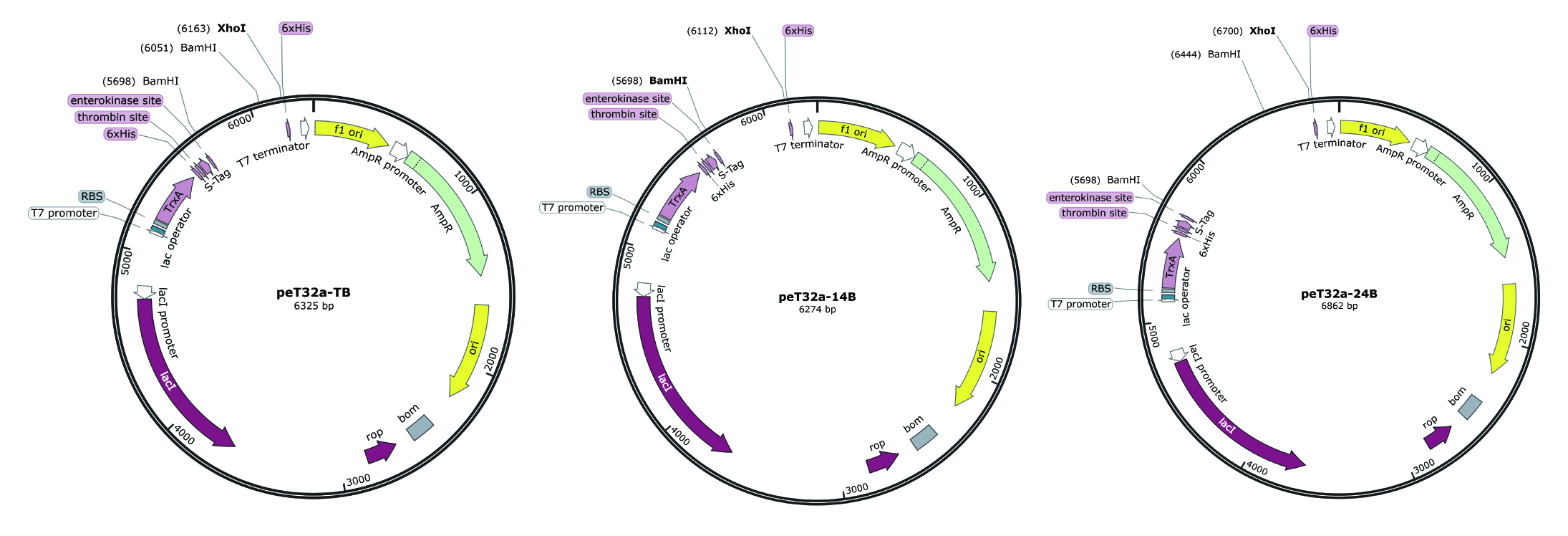


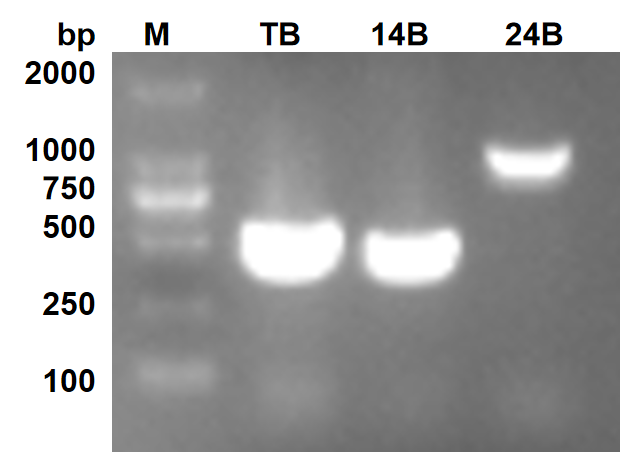

Supplement: Supplementary file 1 — Supporting Information Figure S1. Prediction of transmembrane regions and signal peptides of the six proteins MerC, HuxC, OMP2, TolC, TbpA, and PilB using TMHMM (left) and SignalP5.0 (right) servers. Figure S2. Prediction of the secondary structures and the topologies of the MEVs by PDBsum. (A, C, E) The secondary structures of TB, 14B, and 24B; (B, D, F) the topologies of TB, 14B, and 24B. H1, H2, …, helices; β, beta turn; γ, gamma turn. Figure S3. Molecular docking of the MEVs with swine leukocyte antigen SLA‐DQ. The model with the lowest energy was chosen as the best binding mode. Figure S4. Schematic diagram and construction of plasmid pET32a::TB, pET32a::14B, and pET32a::24B. Table S1. Bacterial strains and plasmids used in this study. Table S2. Experimental groups were used for the evaluation of the immunoprotective effect of the MEVs in mice infection model. Table S3. Prediction of transmembrane regions and signal peptides of the six proteins MerC, HuxC, OMP2, TolC, TbpA, and PilB using TMHMM and SignalP5.0 servers. Table S4. B‐cell epitope prediction using ABCpred and BepiPred‐2.0. Table S5. The final amino acid sequences of TB, 14B, and 24B. The colors of the epitopes and linkers are inconsistent with those in Figure 2. Table S6. Evaluation of the antigenicity, allergenicity, and physical and chemical properties of the MEVs. Table S7. Interface statistics of the docked complexes. [file TBED-2026-5696948-s001.docx]
